# Effects of Traditional Chinese Medicine Shuxuetong Injection on Random Skin Flap Survival in Rats

**DOI:** 10.1155/2014/816545

**Published:** 2014-02-26

**Authors:** Leyi Cai, Wenfang Huang, Dingsheng Lin

**Affiliations:** ^1^Department of Hand and Plastic Surgery, The Second Affiliated Hospital of Wenzhou Medical University, No. 109, XueYuan Road (West), Lucheng, Wenzhou, Zhejiang 325000, China; ^2^Department of Anesthesia, The Children's Hospital Zhejiang University School of Medicine, No. 57 Zhugan Lane, Hangzhou, Zhejiang 310006, China

## Abstract

*Background*. A Shuxuetong injection is traditionally used in Chinese medicine to treat “blood stasis and stagnation” (yu xue yu zhi). We investigated the effect of such injection on the survival of random skin flaps. *Methods*. McFarlane flaps were established in 60 rats divided into two groups. Postoperative celiac injections were given to both groups for 7 days. Shuxuetong was injected into the test group, and saline was injected into controls. On day 7, tissues were stained with H&E (hematoxylin-eosin) stain, immunohistochemically evaluated, and the expression levels of xanthine oxidase were determined. *Result*. The mean area of flap survival in the test group was significantly higher than in controls. Expression of vascular endothelial growth factor and superoxide dismutase, and microvessel development, were markedly increased in the test group, and the malondialdehyde level was reduced. *Conclusion*. Shuxuetong promotes random skin flap survival.

## 1. Introduction

Local skin flaps are often created during plastic and reconstructive surgery to repair tissue defects. However, the length-to-width ratio currently cannot exceed 1.5–2 : 1, greatly limiting the clinical applications of random skin flaps. Previous work [1, 2] has suggested that inadequate blood perfusion, ischemia-reperfusion injury, and expression of apoptosis-related factors contribute directly to flap necrosis. Several pharmacological agents have been used in efforts to prevent or reverse skin flap ischemia. These include vascular endothelial growth factor (VEGF) [3], aspirin [4], and eutectic mixture of local anesthetics (EMLA; a mix of lidocaine and prilocaine) [5].

A Shuxuetong injection is traditionally used in Chinese medicine to treat “blood stasis and stagnation” (yu xue yu zhi), particularly in patients with cerebral and myocardial infarction [6]. The material is extracted from *Hirudo* leeches and also contains *Pheretima* (defined below). Hirudin, an antithrombotic substance produced by the salivary glands of the medicinal leech (*Hirudo medicinalis*), is the most potent and specific direct thrombin inhibitor (DTI) known [7]. Hirudin blocks the thrombin-mediated conversion of fibrinogen to fibrin, thus inhibiting clot formation. Hirudin also inhibits platelet aggregation [8]. *Pheretima* (from the earthworm *Lumbricus bimastus*) contains a thrombolytic enzyme termed lumbrokinase (PI239), which shows powerful antithrombotic and fibrinolytic activities [9]. The enzyme mediates anticoagulation by activating plasminogen, promoting thrombolysis, and preventing thrombus recurrence. Moreover, recent work has shown that Shuxuetong facilitates angiogenesis during wound healing following traumatic brain injury [6]. In addition, the material has a protective effect on brain tissue subject to ischemia-reperfusion injury, by enhancing expression of gamma-aminobutyric acid (GABA) in the hippocampus [10].

Thus, Shuxuetong injection should optimally restore the blood supply and promote wound healing. However, any utility of Shuxuetong for enhancing flap survival remains unclear. In the present work, we explored the effect of Shuxuetong on the survival of random skin flaps and the mechanism thereof.

## 2. Material and Methods

### 2.1. Animal Model and Drug Administration

Sixty male Sprague-Dawley (SD) rats (250–300 g) were obtained from the Wenzhou Medical College (SCXK [Zhe] 2005-0019) and treated in accordance with the Guide for the Care and Use of Laboratory Animals of Wenzhou Medical College. The rats were randomly divided into a Shuxuetong group and a control group (30 rats/group). They were anesthetized with 5% (w/v) chloralic hydras (6–8 mL/kg, intraperitoneally), and then a McFarlane [11] flap (3 × 9 cm) was created on the same position of the dorsum of each rat, using the iliac crest to ensure consistent positioning.

Perforated vessels at the flap bases were ligatured to create completely random vascular patterns. After separating subcutaneous tissue in the deep fascia, each flap was sutured back to the original position using continuous 4–0 silk sutures. Prior to analysis, each flap was divided into three distinct zones of equal size, the proximal area (I), the middle area (II), and the distal area (III) [12, 13]. Aseptic techniques were rigorously applied. All rats were individually housed after surgery to prevent cannibalism or injury caused by normal socialization. All operations were performed by one researcher and no rat died during the procedure.

Commencing immediately after operation, injections of Shuxuetong (catalog number 100403-1, Mu-dan-jiang-you-bo Pharmaceutical Co. Ltd., Mudanjiang, China) were given to test rats (1.5 mL/kg; intraperitoneally for 7 successive days). Control rats received saline. At the end of treatment, flap condition was evaluated in terms of appearance, color, texture, and hair condition. Seven days later, all animals were sacrificed via administration of chloral hydrate, and flap tissues were dissected. Animal care and euthanasia conformed to the principles of the guide mentioned above.

### 2.2. Assessment of Survival Areas

To quantify survival area, flaps were photographed, and surviving areas were measured by superimposition of photographs on graph paper. The results are expressed as percentages of viable area, calculated as follows: extent of viable area × 100/total area (viable and ischemic). Flap tissues were biopsied for histological assessment and immunohistochemical staining for VEGF.

### 2.3. Microvascular Density

Three samples (1 cm × 1 cm) of central flap tissue were collected from each area and fixed in 4% (v/v) paraformaldehyde in PBS for 24 h. Paraffin sections were prepared using a routine technique. Slices (4 *μ*m) were subjected to H&E staining and examined by light microscopy (Olympus BH51, Tokyo, Japan; 100x magnification). We evaluated the thickness of granulation tissue and whether edema and neutrophil infiltration were evident. We measured microvascular density (MVD) as follows. First, we microscopically identified the most vascularized areas under low magnification (40x). Next, we counted vessels (at 400x) in five random microscopic fields each 0.152 mm^2^ in area, thus 0.44 mm in diameter. We calculated microvascularity per unit area (mm^2^) as an indicator of MVD [14]. Generally, single endothelial cells, endothelial cell clusters, and vessels containing less than eight erythrocytes were considered microvessels. All slides were evaluated by two independent observers blinded to each other's findings and to experimental groupings. The mean MVD (from the two examiners) was subjected to statistical analysis.

### 2.4. VEGF Expression

VEGF expression level was evaluated immunohistochemically employing a streptavidin/peroxidase-based protocol. We first blocked slides with normal goat serum at room temperature for 20 min and added 50 *μ*L anti-VEGF antibody solution (diluted 1 : 100). Incubation at 4°C overnight followed. All slices were warmed to 37°C for 45 min and washed with PBS. Next, 50 *μ*L goat anti-rat antibody (diluted 1 : 50) was added, followed by incubation at 37°C for 1 h and rinsing with PBS. For color development, samples were incubated in 3,3 N-diaminobenzidine tetrahydrochloride (DAB) solution for 5 min. The most intensely stained areas were identified under low magnification, and then vessels in five fields of each slice were viewed under higher magnification (400x). Observation parameters (white balance, aperture, shutter speed, and time) were held constant. Images were saved using Image-Pro Plus software, version 6.0 (Media Cybernetics, Rockville, MD) and the integral absorbance (IA) values were used as indicators of VEGF expression levels.

### 2.5. Analysis of Superoxide Dismutase Activity and Evaluation of Malondialdehyde Content

On day 7 postoperatively, 30 tissue specimens (0.5 cm × 0.5 cm) were obtained from the section II/III boundaries, weighed, homogenized, and diluted to 10% (v/v) in an ice bath. Superoxide dismutase (SOD) activity was determined using the xanthine oxidase method, and malondialdehyde (MDA) content was measured by reacting the material with thiobarbituric acid (TBA) at 90–100°C [15].

### 2.6. Statistical Analysis

All results are expressed as means ± SDs. Data were analyzed with the aid of SPSS version 16.0 software. Graphs were constructed using GraphPad Prism version 5.0.

A *P* value <0.05 was considered statistically significant. The extent of necrotic change and histological and immunohistochemical findings were compared using the Mann-Whitney test.

## 3. Results 

### 3.1. Shuxuetong Improves Flap Survival

**Figure 1:**
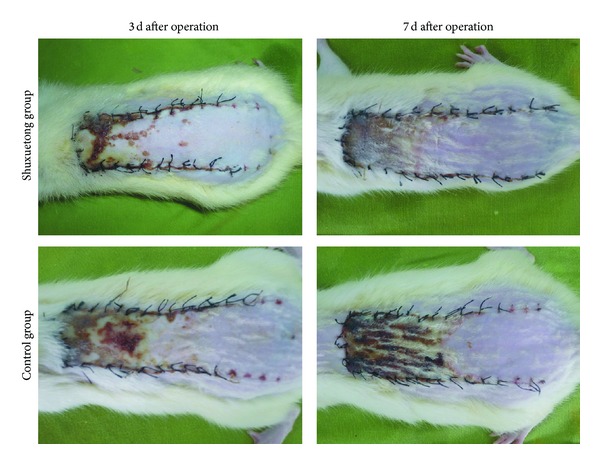
Digital photographs showing the general form of postoperative flaps in the Shuxuetong and control groups. The photographs were taken on days 3 and 7.

The general condition of each flap was recorded daily at the time of injection. On the first day, all flaps were swollen to some extent, and distal area III was dark purple in color, but without obvious necrosis. On the third day, flap areas II and III in both control and experimental groups exhibited reddish-brown focal or patchy necrosis, with congestion. On the seventh day, most of these necrotic parts had fused, scabbed, and hardened. The boundaries between necrotic and surviving regions were stable. Surviving flap portions grew fine hair but the necrotic regions became hard, dark, and glabrous and did not bleed when cut with a scalpel (Figure 1).

The mean surviving area was 72.52 ± 2.23% in the Shuxuetong group, significantly higher than the 50.36 ± 2.37% in the control group (*P* < 0.05).

### 3.2. Shuxuetong Facilitates Angiogenesis

**Figure 2:**
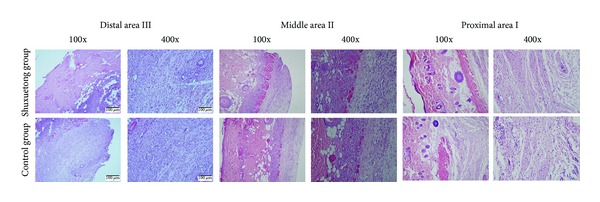
Histological changes in areas I, II, and III of flaps of the Shuxuetong and control groups. Magnifications: 100x and 400x. H&E stain.

Seven days after operation, the distal areas were morphologically similar in histological terms. All flaps exhibited similar changes in appearance; inflammatory cell infiltration was prominent, as were structural damage and edema. Ninety percent of tissue images revealed degeneration and necrosis of muscle fibers. In flap area II, the Shuxuetong group exhibited greater proliferation of fibroblasts, thinner granulation tissue with less edema, more scattered areas of subcutaneous hemorrhage, more diffuse neutrophil infiltration, and more neovascularization, compared to the control group (Figure 2). In area I, the Shuxuetong group exhibited edema, vascular dilation, and inflammatory cell infiltration to a lesser extent than controls.

**Figure 3:**
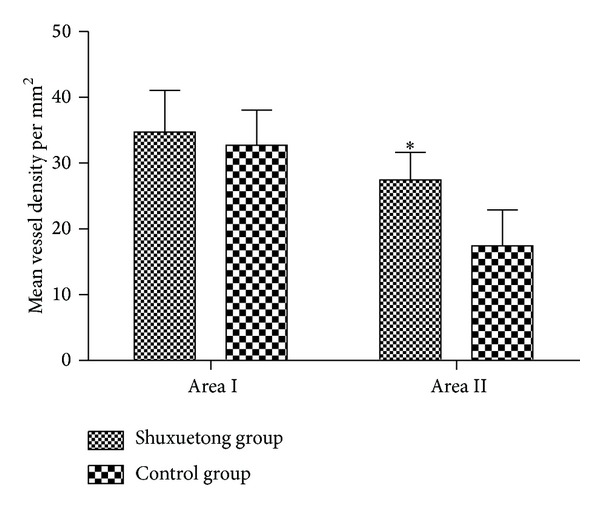
The MVDs of areas I and II of the ShuXueTong and control groups.

The MVDs of area I in the Shuxuetong and control groups were 34.71 ± 6.37/mm^2^ and 32.71 ± 5.36/mm^2^, respectively, (Figure 3), thus not significantly different (*P* > 0.05). The respective MVDs of area II were 27.42 ± 4.21/mm^2^ and 17.45 ± 5.43/mm^2^(Figure 3). This difference was statistically significant (*P* < 0.05).

### 3.3. VEGF Expression by Endothelial Cells Is Enhanced by Shuxuetong

**Figure 4:**
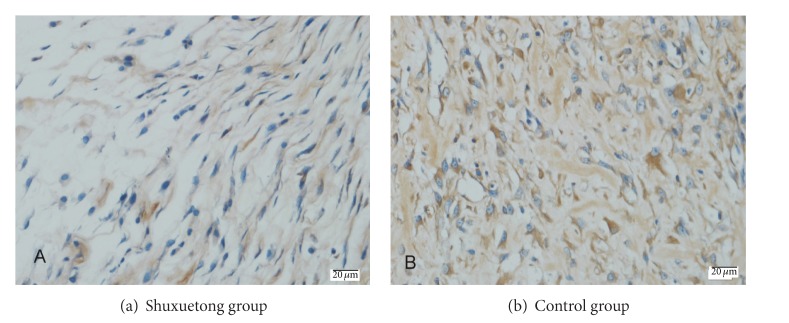
VEGF protein levels in the Shuxuetong and control groups. (immunohistochemical staining) (×400).

A significant (*P* < 0.05) difference in VEGF expression was evident between the two groups as revealed by IA data (Figure 4). The expression level in the Shuxuetong group was 4,731.24 ± 448.99 IA and that of the control group was 2,466.01 ± 801.67.

### 3.4. Shuxuetong Protects Flap against Ischemia-Reperfusion Injury

**Figure 5:**
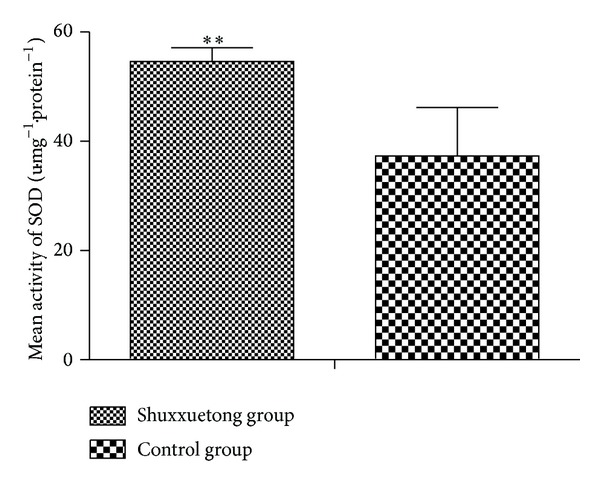
SOD activities in the Shuxuetong and control groups.

**Figure 6:**
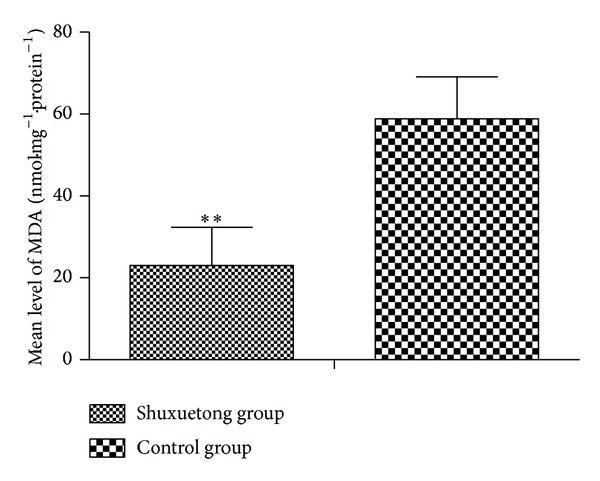
MDA levels in the Shuxuetong and control groups.

The mean SOD activity in the Shuxuetong group was 54.633 ± 2.497 units/mg protein, significantly higher than that in the control group (37.34 ± 8.842; *P* < 0.05) (Figure 5). However, the mean MDA level in the experimental group (23.028 ± 9.357 nmol/mg protein) was significantly less than that in the control group (58.810 ± 10.290) (*P* < 0.05) (Figure 6).

## 4. Discussion

The components of Shuxuetong are derived from the leech and the earthworm. In Chinese traditional medicine, both components promote blood flow (huo xue), activate meridians (tong luo), and disperse blood stasis (hua yu) [6]. Thus, we logically hypothesized that Shuxuetong might facilitate angiogenesis.

The role played by VEGF in initiating proliferation of endothelial cells has been well documented. VEGF promotes revascularization of ischemic flaps, thus increasing flap survival [16, 17]. Moreover, Shuxuetong injections enhance VEGF/VEGFR-2 expression in patients with traumatic brain injuries [6, 18]. In the present study, the VEGF expression level was markedly higher in test flaps than in controls. Furthermore, the MVD in area II of the Shuxuetong group was significantly greater than that in controls. These results suggest that Shuxuetong may promote neovascularization and microcirculation in ischemic flaps by increasing VEGF expression, ultimately improving flap viability. The detailed mechanism requires further study.

Generation of oxygen-derived free radicals contributes greatly to the pathogenesis of ischemia-reperfusion injury, and the topic has been well studied. A burst of free oxygen radicals attacks lipids of the cell membrane as well as membrane proteins within the first few minutes of reperfusion. In addition, reperfusion leads to the accumulation of activated neutrophils in ischemic tissue and activation of xanthine oxidase in endothelial cells, causing rapid necrosis of the flap [15, 19].

SOD, metalloprotein, is an important antioxidase that inactivates O^−2^ (the predecessor of H_2_O_2_ and OH^−^), thus protecting cells from injury by toxic oxygen-derived free radicals. Such radicals cause peroxidation of lipids and proteins and damage cell and organelle membranes, thus destroying tissue structure and function. MDA is one product of lipid peroxidation, and the MDA level thus indirectly reflects the extent of tissue damage [20]. In the present study, we found that SOD activity, MDA level, and the surviving flap area were significantly better in the test group than in controls. We thus confirmed that Shuxuetong had a protective effect in terms of endogenous SOD activity and inhibited lipid peroxidation. It also inhibited the development of ischemic reperfusion injury by increasing free radical scavenging.

The antithrombotic, anticoagulant, and fibrinolytic activities of Shuxuetong may also contribute to the survival of random skin flaps.

## 5. Conclusions

Shuxuetong injection should be considered potentially as a choice for microsurgery. Undoubtedly, this medicine shows an excellent result for the survival of skin random flap.
